# Radiative Hydromagnetic Flow of Jeffrey Nanofluid by an Exponentially Stretching Sheet

**DOI:** 10.1371/journal.pone.0103719

**Published:** 2014-08-01

**Authors:** Tariq Hussain, Sabir Ali Shehzad, Tasawar Hayat, Ahmed Alsaedi, Falleh Al-Solamy, Muhammad Ramzan

**Affiliations:** 1 Department of Mathematics, Faculty of Computing, Mohammad Ali Jinnah University, Islamabad Campus, Islamabad, Pakistan; 2 Department of Mathematics, Quaid-i-Azam University, Islamabad, Pakistan; 3 Nonlinear Analysis and Applied Mathematics (NAAM) Research Group, Faculty of Science, King Abdulaziz University, Jeddah, Saudi Arabia; Northwestern Polytechnical University, China

## Abstract

Two-dimensional hydromagnetic flow of an incompressible Jeffrey nanofluid over an exponentially stretching surface is examined in the present article. Heat and mass transfer analysis is performed in the presence of thermal radiation, viscous dissipation, and Brownian motion and thermophoresis effects. Mathematical modelling of considered flow problem is developed under boundary layer and Rosseland’s approximations. The governing nonlinear partial differential equations are converted into ordinary differential equations via transformations. Solution expressions of velocity, temperature and concentration are presented in the series forms. Impacts of physical parameters on the dimensionless temperature and concentration are shown and discussed. Skin-friction coefficients are analyzed numerically. A comparison in a limiting sense is provided to validate the present series solutions.

## Introduction

Boundary layer flow over a stretched surface is a subject of abundant studies now a days because of its existence in various engineering and industrial processes like cooling of metallic sheets in a cooling bath, annealing and thinning of copper wires, aerodynamic extrusion of plastic and rubber sheets, drawing of plastic films and sheets, glass fiber and paper production etc. It is worthmentioning to point out here that the stretching velocity is not linear necessarily in all the cases. The stretching velocity may be nonlinear or exponential. For example in annealing and thinning of copper wires, the desired quality product depends on the continuous stretching of surface with exponential dependence velocity distribution [1]–[6]. The magnetohydrodynamic flow is quite interesting in engineering and industrial processes. El Koumy et al. [7] investigated peristaltic flow of Maxwell fluid through a porous medium in the presence of Hall effects. Hall currents and heat transfer analysis in peristaltic flow is performed by Abo-Eldahab et al. [8]. Effects of magnetic field and porous space in peristaltic flow of Maxwell fluid are examined by Mekheimer et al. [9]. Shehzad et al. [10] discussed the Joule heating and thermophoresis effects in MHD flow of Jeffrey fluid induced by a stretching sheet. An induced magnetic field and slip effects in peristaltic flow through a porous medium are described by Mekheimer et al. [11]. Recently, Hayat et al. [12] performed a study to analyze the effects of Hall current and Ohmic heating in peristaltic flow of non-Newtonian fluid.

A working fluid is involved in many engineering and industrial applications that are in flowing or stagnant state. This working fluid is used to transfer energy/heat from one position to the other. The adequate heat transfer performance has been a major issue for a long period. This issue can be resolved by using a new working fluid that has better thermal performance than the ordinary base liquids. Recently nanofluid is the best candidate to take place of working fluid. Nanofluid is a fluid in which the nanoparticles are submerged. The size of these nanoparticles is 1–100 nm. The thermal conductivity of the nanofluids is higher than that of base fluids. Further, the novel properties of Brownian motion and thermophoresis of such fluids make them potentially useful. Nanoparticles are used to enhance the thermal characteristics of ordinary base fluids such as water, ethylene glycol or oil [13]. In addition the magneto-nanofluid is a unique material that has both liquid and magnetic properties. Such nanofluid has superficial role in construction of loud speakers, blood analysis and cancer therapy. Buongiorno [14] provided a mathematical model of nanofluid which has the characteristics of thermophoresis and Brownian motion. Later on, Makinde and Aziz [15] investigated the boundary layer flow of viscous nanofluid with convective thermal boundary condition. Here, the flow is induced due to linear stretching of surface. Closed form solutions of MHD nanofluid flow with heat and mass transfer analysis in the presence of slip condition were developed by Turkyilmazoglu [16]. Ibrahim and Makinde [17] analyzed the effects of thermal and concentration stratifications in boundary layer nanofluid flow by a vertical surface. Second law of thermodynamics in MHD steady flow of nanofluid over a rotating disk was discussed by Rashidi et al. [18]. Moradi et al. [19] presented the series solutions of Jeffrey Hamel flow by considering the different types of nanoparticles. Unsteady natural convection flow of nanofluid with heat and mass transfer over a vertical plate was examined by Turkyilmazoglu and Pop [20]. Slip and Joule heating effects in MHD peristaltic flow of nanofluid under thermal diffusion and diffusion thermo effects was studied by Hayat et al. [21]. Sheikholeslami et al. [22] carried out an analysis to discuss the effects of an applied magnetic field in rotating flow of nanofluid with heat transfer.

The aim of present article is to study the flow analysis of Jeffrey fluid [23]–[28] in the presence of thermophoresis, Brownian motion, thermal radiation and viscous dissipation effects. The flow caused is by an exponentially stretching sheet. Jeffrey fluid has ability to exhibit the properties of ratio of stress relaxation to retardation and retardation. Mathematical formulation is performed under boundary layer and Rosseland’s assumptions. Homotopy analysis method (HAM) [29]–[35] is utilized for solution expressions of dimensionless velocity, temperature and concentration. Temperature and concentration fields are shown and discussed for the different values of arising parameters. Skin-friction coefficients are computed and analyzed. Comparison of local Nusselt number in a limiting sense is tabulated and analyzed.

## Mathematical Model

We consider the two-dimensional hydromagnetic flow of Jeffrey nanofluid over an exponentially stretching sheet. Heat and mass transfer effects are taken into account. An applied magnetic field of strength 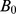 is encountered normal to the flow direction. The magnetic Reynolds number is chosen small and the Joule heating effects are absent. Further the induced magnetic field is smaller in comparison to the applied magnetic field and is negligible. In addition, viscous dissipation effects are also taken into account. The two-dimensional boundary layer equations of an incompressible Jeffrey nanofluid with heat and mass transfer are given below:
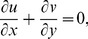
(1)

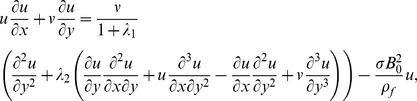
(2)

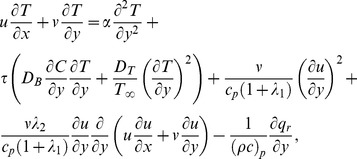
(3)

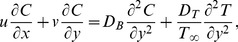
(4)


The boundary conditions for the considered flow analysis are
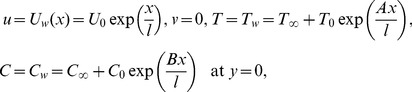
(5)

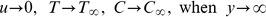
(6)where 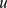 and 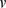 are the velocity components in the 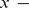 and 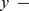
directions, 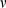 the kinematic viscosity, 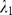 the ratio of relaxation to retardation times, 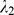 the relaxation time, 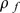 the density of fluid, 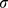 the Steffan-Boltzman constant, 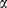 the thermal diffusivity, 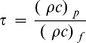 the ratio of nanoparticle heat capacity and the base fluid heat capacity, 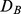 the Brownian diffusion coefficient, 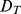 the thermophoretic diffusion coefficient, 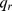 the radiative heat flux, 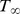 and 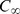 are the ambient fluid temperature and concentration far away from the sheet and 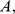 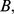 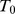, 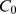 are the constants.

By employing Rosseland's approximation, Eq. (3) has the form
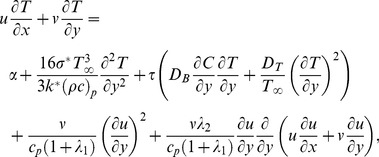
(7)


The dimensionless variables are defined as
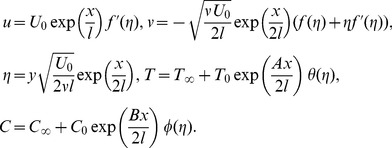
(8)


The equations of linear momentum, energy, concentration and their corresponding boundary conditions in dimensionless form can be written as
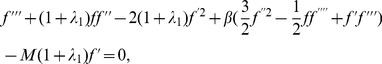
(9)

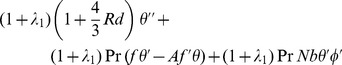


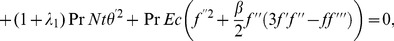
(10)

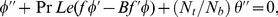
(11)

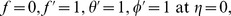


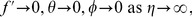
(12)where 
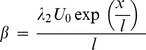
 is the Deborah number, 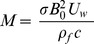 is the magnetic parameter, 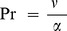 is the Prandtl number, 
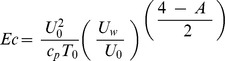
 is the Eckert number, 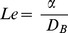 is the Lewis number, 
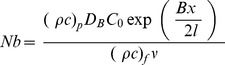
 is the Brownian motion parameter, 
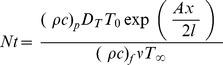
 is the thermophoresis parameter,

The skin friction coefficient, the local Nusselt and Sherwood numbers are defined below.
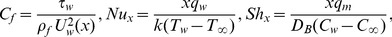
(13)where 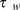 is the shear stress along the stretching surface, 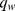 is the surface heat flux and 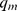 is the surface mass flux. The local skin-friction coefficient, local Nusselt and Sherwood numbers in dimensionless forms can be written as
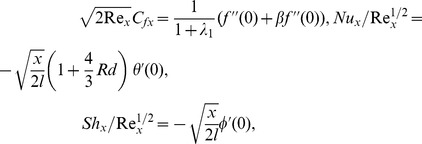
(14)where 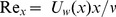 is the local Reynolds number.

### Homotopy Analysis Solutions

To proceed the homotopic solutions, the initial guesses and auxiliary linear operators are chosen as follows:
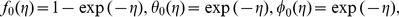
(15)

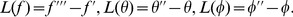
(16)


The above initial guesses and auxiliary linear operators satisfies the below mentioned properties
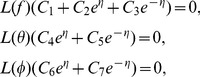
(17)where 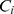 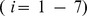 are the arbitrary constants.

The zeroth order problems can be written as
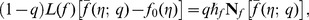
(18)

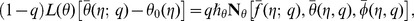
(19)

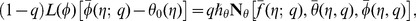
(20)

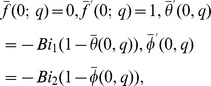


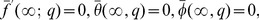
(21)

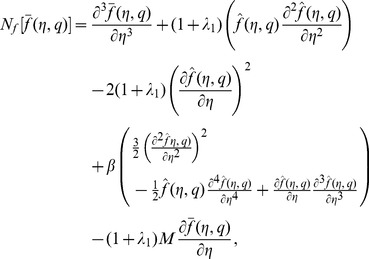
(22)

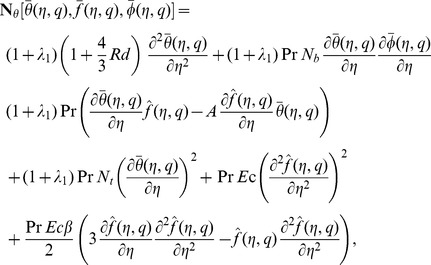
(23)

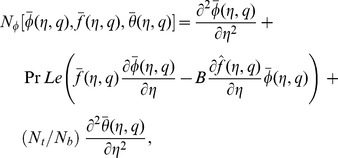
(24)where 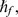 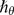 and 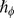 are the non-zero auxiliary parameters, 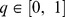 is an embedding parameter and 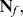 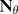 and 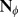 are the nonlinear operators. Putting 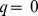 and 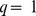 one has
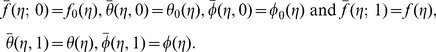
(25)When we increase the values of 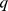 from 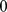 to 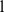 then 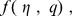 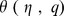 and 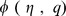 vary from 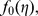 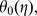 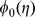 to 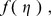 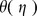 and 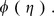 By adopting Taylor series expansion, we have [28]–[30]:



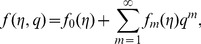
(26)

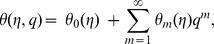
 (27)
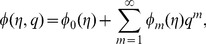
(28)

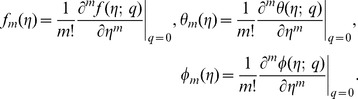
(29)


The convergence of above series highly depends upon the suitable values of 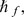 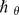 and 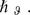 Considering that 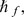 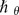 and 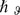 are selected properly such that (26)–(28) converge at 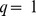 and then we have
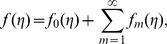
(30)

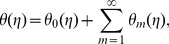
(31)

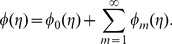
(32)


The general solutions can be written as
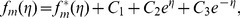
(33)

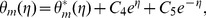
(34)

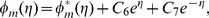
(35)where 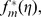 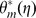 and 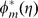 are the special solutions.

## Convergence Analysis and Discussion

**Figure 1:**
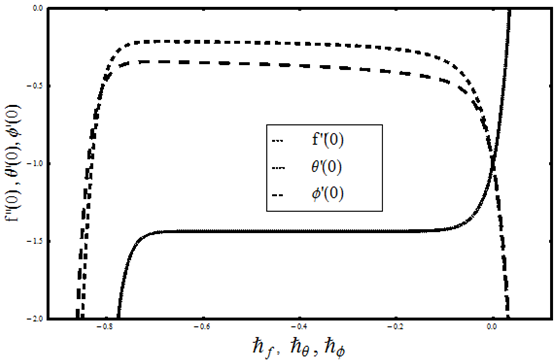
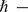
curves for functions 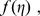 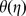 and 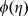 at 21th order of approximations when 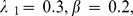 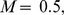 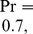 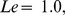 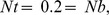 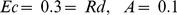 and 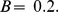

The auxiliary parameters 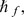 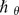 and 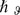 are encountered when homotopy analysis method has been utilized to compute the series solutions. These parameters have essential importance for adjusting and controlling the convergence of series solutions. The appropriate values of these parameters are required for the convergent solutions. To obtain the proper values of these auxiliary parameters, we drawn the 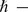
curves at 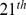-order of HAM approximations. These 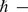
curves are presented in Fig. 1. From this Fig. we examined that the suitable values of 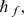 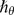 and 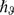 are 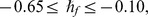 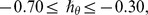 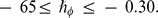 The series converges in the whole region of 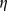 when 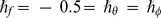 (see Table 1).

**Table 1:** Convergence of homotopy solution for different order of approximations when 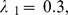 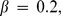 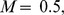 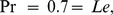 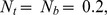 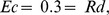 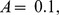 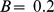 and 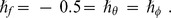

Order ofapproximation	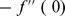	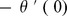	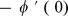
1	1.2900	0.5316	0.5572
5	1.4310	0.2972	0.3806
11	1.4350	0.2433	0.3146
20	1.4350	0.2210	0.2692
30	1.4350	0.2124	0.2435
45	1.4350	0.2076	0.2225
50	1.4350	0.2076	0.2193
60	1.4350	0.2076	0.2193

**Figure 2:**
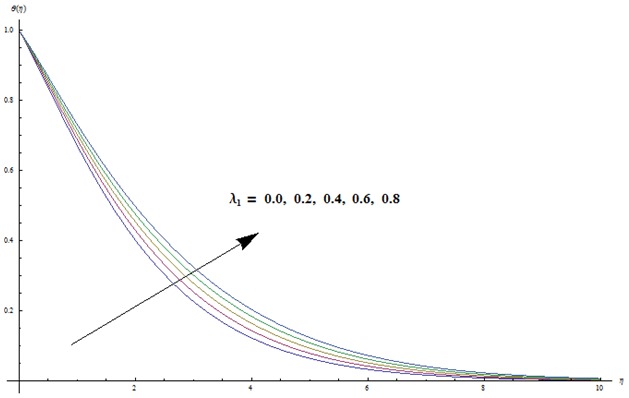
Variation in dimensionless temperature 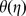 vs 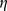 for different values of 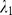 when 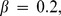 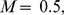 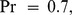 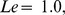 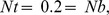 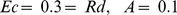 and 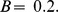

**Figure 3:**
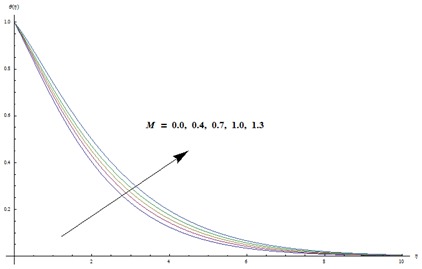
Variation in dimensionless temperature 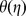 vs 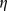 for different values of 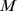 when 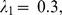 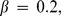 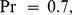 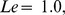 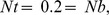 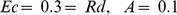 and 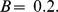

**Figure 4:**
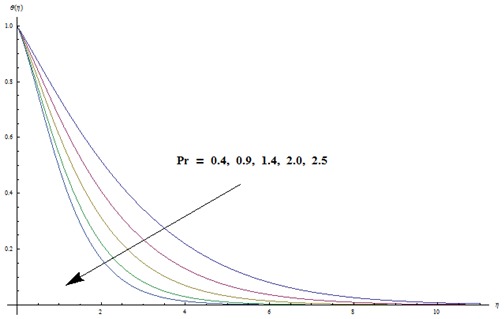
Variation in dimensionless temperature 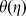 vs 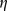 for different values of 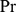 when 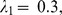 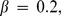 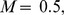 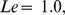 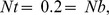 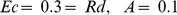 and 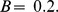

**Figure 5:**
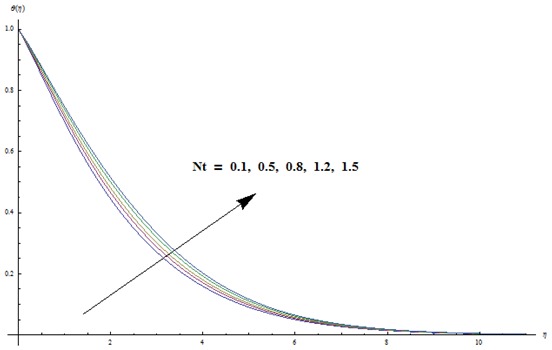
Variation in dimensionless temperature 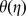 vs 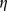 for different values of 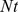 when 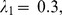 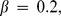 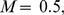 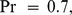 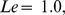 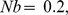 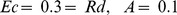 and 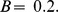

**Figure 6:**
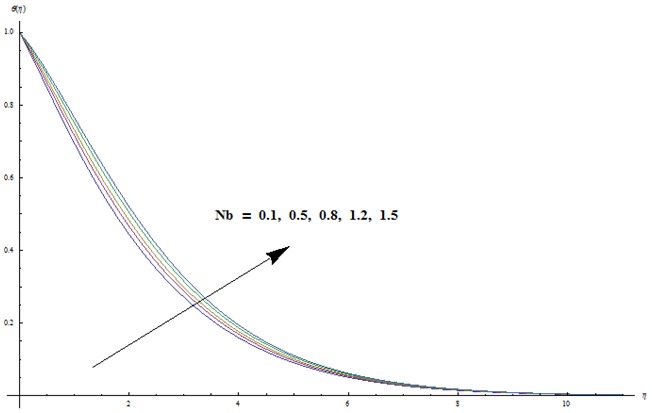
Variation in dimensionless temperature 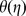 vs 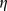 for different values of 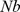 when 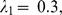 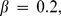 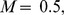 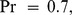 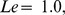 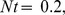 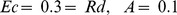 and 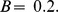

**Figure 7:**
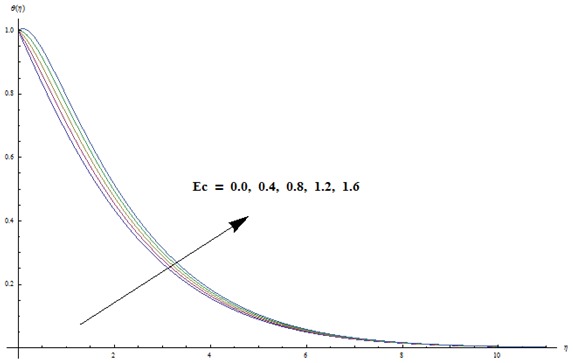
Variation in dimensionless temperature 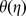 vs 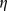 for different values of 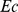 when 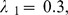 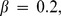 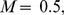 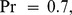 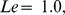 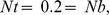 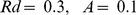 and 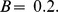

**Figure 8:**
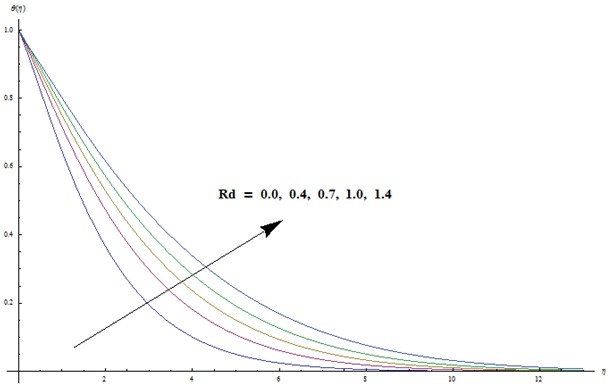
Variation in dimensionless temperature 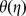 vs 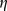 for different values of 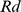 when 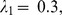 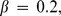 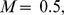 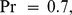 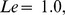 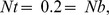 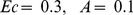 and 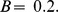

Figs. 2–8 are drawn to examine the variations in dimensionless temperature profile 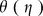 for different values of ratio of relaxation to retardation times 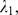 magnetic parameter 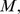 Prandtl number 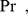 thermophoresis parameter 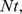 Brownian motion parameter 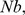 Eckert number 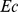 and radiation parameter 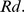 Fig. 2 shows that an increase in ratio of relaxation to retardation times gives rise to the temperature and thermal boundary layer thickness. Minimum temperature and thinnest thermal boundary layer thickness is noticed when 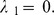 An increase in 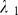 implies to an increase in relaxation time and decrease in retardation time. This change in relaxation and retardation times elucidates the higher temperature and thicker thermal boundary layer thickness. Change in temperature profile for different values of magnetic parameter is noticed in Fig. 3. Here we examined that both temperature and thermal boundary layer thickness are enhanced for larger magnetic parameter. It is also seen that for 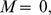 hydrodynamic flow situation is recovered. Magnetic parameter involves the Lorentz force. Higher magnetic parameter implies to stronger Lorentz force and lower magnetic parameter has weaker Lorentz force. Here the stronger Lorentz force leads to an increase in the temperature and thermal boundary layer thickness. Fig. 4 depicts the impact of Prandtl number on the temperature field. It is observed that lower temperature and thinner thermal boundary layer thickness appear corresponding to the increasing values of Prandtl number. Prandtl number is the ratio of viscous to thermal diffusivities. Larger Prandtl number fluids have higher viscous diffusivity and weaker thermal diffusivity. Such change in viscous and thermal diffusivities leads to a decrease in thermal boundary layer thickness. Figs. 5 and 6 illustrate that both temperature and thermal boundary layer thickness are enhanced when the larger values of thermophoresis and Brownian motion parameters are taken into account. From Fig. 7 we noticed that temperature is an increasing function of Eckert number. Further 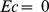 corresponds to the analysis when viscous dissipation effects are absent. Effects of radiative parameter on the temperature field are analyzed in Fig. 8. We observed that an increase in radiative parameter leads to an enhancement in the temperature field. In fact an increase in radiative parameter provides more heat to fluid that corresponds to higher temperature.

**Figure 9:**
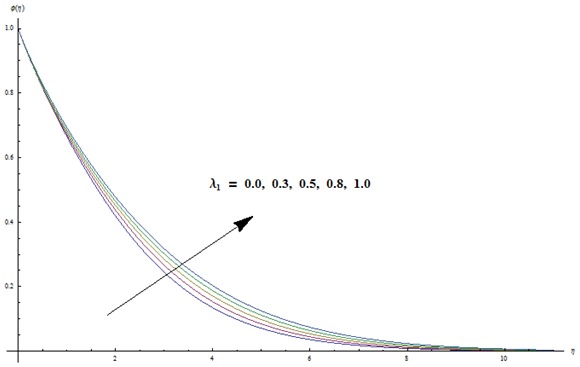
Variation in dimensionless concentration 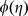 vs 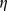 for different values of 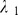 when 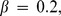 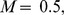 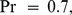 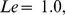 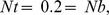 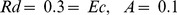 and 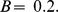

**Figure 10:**
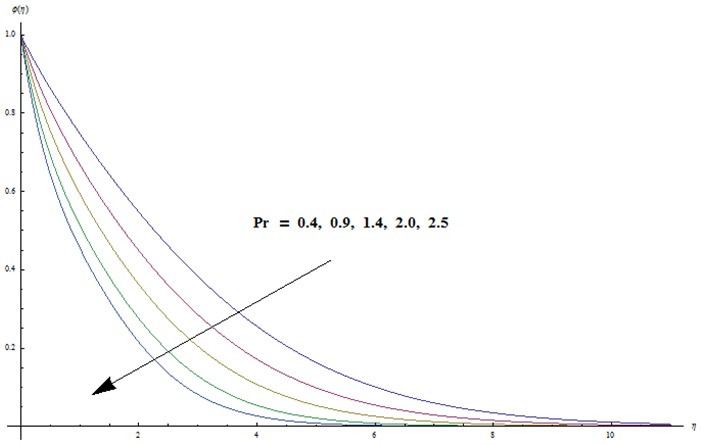
Variation in dimensionless concentration 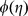 vs 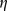 for different values of 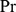 when 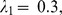 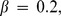 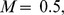 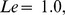 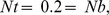 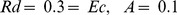 and 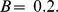

**Figure 11:**
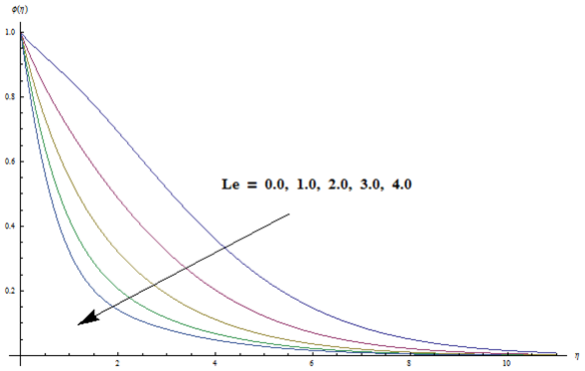
Variation in dimensionless concentration 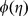 vs 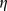 for different values of 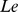 when 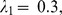 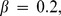 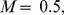 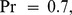 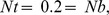 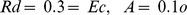 and 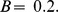

**Figure 12:**
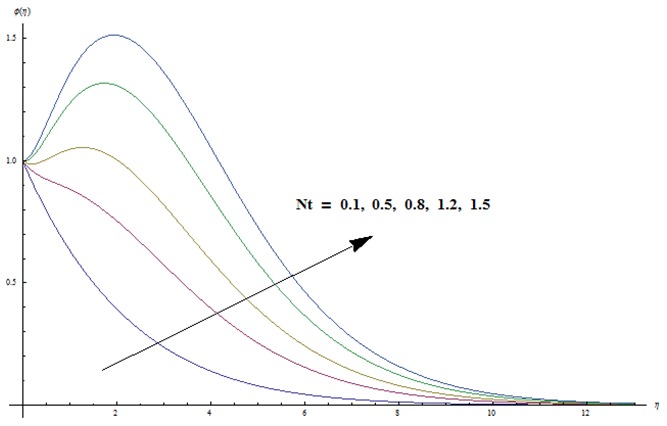
Variation in dimensionless concentration 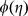 vs 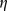 for different values of 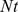 when 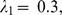 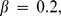 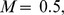 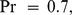 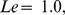 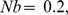 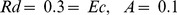 and 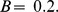

**Figure 13:**
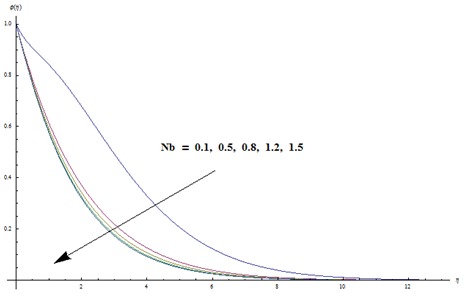
Variation in dimensionless concentration 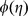 vs 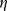 for different values of 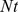 when 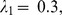 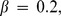 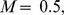 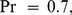 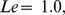 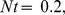 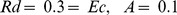 and 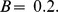

**Figure 14:**
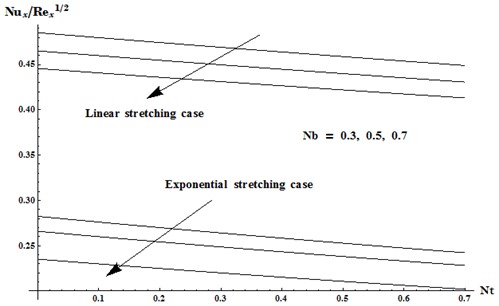
Variation in Nusselt and Sherwood numbers for different values of 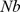 vs 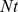 when 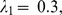 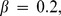 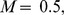 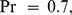 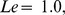 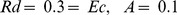 and 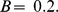

**Figure 15:**
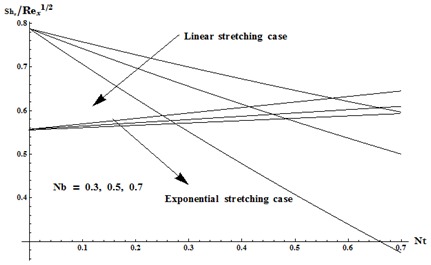
Variation in Nusselt and Sherwood numbers for different values of 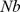 vs 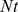 when 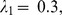 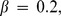 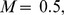 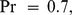 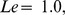 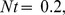 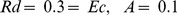 and 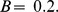

Change in concentration distribution function 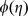 for various values of ratio of relaxation to retardation times 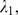 Prandtl number 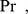 Lewis number 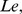 thermophoresis parameter 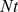 and Brownian motion parameter 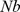 is examined through the Figs. 9–13. Fig. 9 clearly indicates that concentration and its related boundary layer thickness are increasing functions of ratio of relaxation to retardation times. In addition a comparison of the Figs. 2 and 9 shows that the impacts of ratio of relaxation to retardation times on the temperature and concentration are quite reverse. Fig. 10 depicts that concentration boundary layer thickness become thinner for larger Prandtl number. Form Fig. 11 we noticed that an increase in Lewis number shows lower concentration field. Lewis number is inversely proportional to the Brownian diffusion coefficient. This Brownian diffusion coefficient becomes smaller corresponding to the larger values of Lewis number. This smaller Brownian diffusion coefficient shows a reduction in the concentration and associated boundary layer thickness. Fig. 12 presents that the larger values of thermophoresis parameter give rise to the concentration and its related boundary layer thickness. It is also seen that concentration is at the peak when 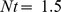 and 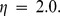 Fig. 13 is drawn to see the variations in concentration profile when 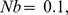 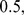 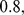 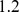 and 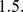 We have seen that the concentration and its related boundary layer thickness is decreased by increasing the Brownian motion parameter. It is also observed that the concentration profile decreases rapidly when 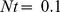 but after 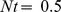 this reduction is very small (see Fig. 13). Figs. 14 and 15 are drawn to examine the variations in Nusselt and Sherwood numbers for different values 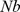 vs 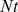 for both linear and exponential stretching cases. Here we noticed that heat and mass transfer rates at wall are higher for linear stretching case in comparison to exponential case.

Table 1 is computed to examine the values of 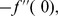 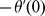 and 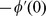 when 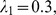 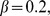 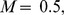 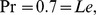 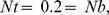 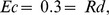 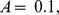 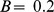 and 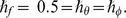 From this Table, we analyzed that the values of 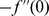 converge from 11th order of HAM approximations whereas the values of 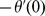 and 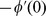 converge from 45th and 50th order of HAM deformations, respectively. Numerical values of skin-friction coefficient 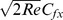 for different values of 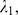 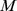 and 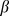 of both exponential stretching and linear stretching cases are presented in Table 2. Here we examined that the values of skin-friction coefficient are smaller for larger 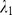 while these values are increased when larger values of 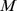 and 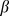 are encountered. Further, it is noticed that the values of skin-friction coefficient are larger for exponential stretching case in comparison to the linear stretching case. Table 3 provides a comparison study with the existing solutions for different values of 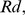 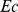 and 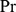 when 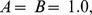 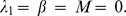 From this Table one can see that our present results have an excellent agreement with the results of Bidin and Nazar [3].

**Table 2:** Numerical values of skin friction coefficient 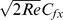 for different values of 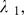 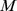 and 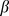 in case of exponential and linear stretching.

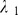	*M*	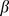	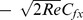	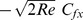
			Exponentialstretching case	Linearstretching case
0.0	0.5	0.2	1.63612	1.34164
0.4			1.38278	1.13389
0.7			1.25485	1.02899
1.0			1.15689	0.94868
0.3	0.0	0.2	1.25661	0.96077
	0.7		1.50007	1.25269
	1.0		1.59258	1.35873
	1.4		1.70796	1.48842
0.3	0.5	0.0	1.28616	1.07417
		0.3	1.50353	1.22474
		0.5	1.63159	1.31559
		0.8	1.80611	1.44115

**Table 3:** Comparison values of 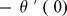 with Bidin and Nazar [3] for different values of 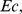 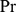 and 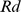 when 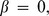 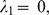 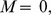 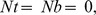 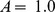 and 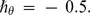.

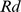	Bidin and Nazar [3]			Present HAM solutions		
	*Ec = 0.0*			*Ec = 0.0*		
	*Pr = 1.0*	*Pr = 2.0*	*Pr = 3.0*	*Pr = 1.0*	*Pr = 2.0*	*Pr = 3.0*
0.0	0.955	1.471	1.869	0.9548	1.4715	1.8693
0.5	0.677	1.074	1.381	0.6775	1.0734	1.3807
1.0	0.532	0.863	1.121	0.5337	0.8627	1.1213
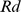	*Ec = 0.2*			*Ec = 0.2*		
	0.862	1.306	1.688	0.8624	1.3055	1.6384
	0.618	0.965	1.229	0.6183	0.9653	1.2286
	0.488	0.782	1.007	0.4889	0.7818	1.0067
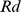	*Ec = 0.9*			*Ec = 0.9*		
	0.539	0.725	0.830	0.5387	0.7248	0.8302
	0.410	0.587	0.696	0.4111	0.5870	0.6963
	0.334	0.498	0.606	0.3355	0.4984	0.6055

## Conclusions

Radiative hydromagnetic flow of Jeffrey nanofluid over an exponentially stretching sheet is studied. Heat and mass transfer phenomena are discussed in the presence of viscous dissipation. The main observations of present investigation are written below:

- Temperature and concentration profiles are enhanced with an increase in the ratio of relaxation to retardation times.
- Larger values of Deborah number 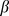 correspond to a reduction in the temperature and concentration fields.
- Thermophoresis and Brownian motion parameters give rise to the temperature and thermal boundary layer thickness.
- Temperature and thermal boundary layer thickness are increasing functions of radiative parameter 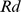 and Eckert number 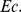

- Brownian motion parameter has reverse effects on temperature and concentration.
- Values of skin-friction coefficient are increased by increasing 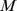 and 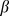 but it decreases when we increase the values of 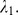
